# A model for Vision Zero implementation in Iran: a grounded theory study

**DOI:** 10.5249/jivr.v14i1.1629

**Published:** 2022-01

**Authors:** Hamid Safarpour, Davoud Khorasani-Zavareh, Hamid Soori, Zohreh Ghomian, Kamran Bagheri Lankarani, Reza Mohammadi

**Affiliations:** ^*a*^ Non-Communicable Diseases Research Center, Ilam University of Medical Sciences, Ilam, Iran.; ^*b*^ Department of Nursing, School of Nursing and Midwifery, Ilam University of Medical Sciences, Ilam, Iran.; ^*c*^ Workplace Health Promotion Research Center (WHPRC), Department of Health in Disasters and Emergencies, School of Public Health and Safety, Shahid Beheshti University of Medical Sciences, Tehran, Iran.; ^*d*^ Safety Promotion and Injury Prevention Research Center, Shahid Beheshti University of Medical Sciences, Tehran, Iran.; ^*e*^ Department of Health in Disasters and Emergencies, School of Public Health and Safety, Shahid Beheshti University of Medical Sciences, Tehran, Iran.; ^*f*^ Health Policy Research Center, Institute of Health, Shiraz University of Medical Sciences, Shiraz, Iran.; ^*g*^ Department of Neurobiology, Care Sciences and Society (NVS), H1, Division of Family Medicine and Primary Care, Karolinska Institutet, Huddinge, Sweden.

**Keywords:** Vision Zero, Road safety, Road traffic crashes, Injury, Qualitative study

## Abstract

**Background:**

Road Traffic injuries (RTIs) are major global health issues, but they have been neglected. RTIs are multi-faceted in nature and, like many injuries, are costly but preventable. Iran has one of the highest rates of deaths due to traffic accidents among middle-income countries. Hence, there is a need for effective and preventive approaches in road safety management. One of the new approaches to road safety is the Vision Zero. The aim of this study was to design a Vision Zero implementation model in Iran.

**Methods:**

This present study was conducted using the qualitative grounded theory approach. Purposive, snowball and maximum variety sampling were used to select participants. In-depth interviews were used to collect data. Grounded theory method was used to analyze the data using Corbin and Strauss method.

**Results:**

In this study, 19 interviews were conducted with 17 participants. Based on data analysis, a total of 4 main categories and 13 subcategories were obtained. According to the participants, the lead agency was recognized as the core category. Other concepts were categorized as causal conditions, intervening conditions, contextual conditions, action/interaction strategies, and consequences.

**Conclusions:**

Establishing a lead agency with inter-organizational coordination through political support and legislation and changing the approach of road safety can be effective in implementing a Vision Zero. Also, improving the safety attitude of the stakeholders and changing their approach through training and advocacy from various organizations related to road safety is effective in creating a lead agency and implementing a vision zero. In addition, in order to implementation of the model, it is very important to pay attention to the economic, political and ethical underlying factors towards human beings.

## Introduction

Transportation system is one of the essential elements of sustainable development of any country. Lack of attention to the improvement of the road safety system can impose many socio-economic damages on countries and threaten the lives of many people.^1^ Road traffic injuries (RTIs) are a major, but neglected, global public health problem.^2^ Road Traffic Crashes (RTCs) are a major cause of death and disability worldwide. Each year, 1.35 million people are killed in RTCs and between 20 and 50 million are injured. RTCs are the most leading cause of death in low- and middle-income countries (LMICs). More than 90% of fatalities due to RTCs occur in LMICs.^3,4^

Iran is one of the countries with the highest rate of deaths caused by RTCs among the Middle East and has the third highest RTIs mortality among higher-middle income countries in the world.^3,5^ According to the report of the Legal Medicine Organization of Iran in the first eight months of 1400 (March 21 to November 22, 2021), about 10,774 people lost their lives due to traffic collisions, which is an increase of about 9.3% compared to the same time in 1399(2020).^6^ RTCs hold a multi-faceted nature and, like many injuries, are costly but preventable. Hence, there is a need for effective and preventive approach in road safety.

One of the new approaches to road safety is the Vision Zero.^4^ Vision Zero is a long-term philosophy and guide in the structure of road safety.^4,7^ In general, the Vision Zero approach is based on four principles of ethics; shared responsibility; safety philosophy; and mechanisms of changes.^8-11^ Vision Zero's long-term goal is that no one should be killed or seriously injured in a traffic accident.^7^ Vision Zero is being considered or implemented in many countries. Since the implementation of Vision Zero, many countries have adopted and executed this approach or significant parts of it, nationally, regionally or locally.^12-17^ Sweden has reduced the number of traffic accidents’ fatality by more than 50% over the last 20 years by implementing the Vision Zero approach. This success is the result of about 70 years of effort to implement Road safety approach.^18-20^ Given the importance of the Vision Zero approach and the results of its implementation in leading countries such as Sweden, it seems that more countries are seeking to implement this approach to reduce traffic accidents. Evidence suggests that no Asian country has yet fully implemented Vision Zero, although countries such as India have tried to implement this approach in road safety in some cities.^21^

Iran has one of the highest rates of mortality caused by RTCs among middle-income countries. Therefore, basic measures are necessary to deal with this issue.^3,22^ However, measures to prevent and reduce deaths and injuries caused by RTCs has been carried out by the Iran's traffic police in 2005, like the accomplishment of a preventive intervention program for RTCs, which contain four measures: requirement of fastening the seat belt, requirement of using of motorcycle helmets, obligation to comply with public traffic laws as well as social media training campaigns;^23^ Yet the evidence shows that these measures have not been effective enough.^24^ Due to this issue and many problems in the transportation system of Iran, including the exhaustion of the transport fleet, the problems of the automotive industry and challenges related to vehicle safety, as well as the lack of full responsibility of traffic accidents’ organizations to their duties,^25^ it seems that a systemic approach should be taken to traffic accidents.

Therefore, considering that the principles of Vision Zero have both a systemic approach and take into account the mistakes and weaknesses of human beings as well as the dimension of ethics and shared responsibility, and can be implemented in any country with any degree of economic income and level of development, so the approach is suitable for preventing deaths and injuries caused by RTCs in Iran. Also, the successful experience of Vision Zero shows that achieving a reduction in mortality due to RTCs in Iran requires the implementation of a successful approach such as the Vision Zero. In order to implement this approach in Iran, first it is necessary to design its implementation model. As a result, this study aimed to design a Vision Zero implementation model in Iran.

## Methods 

**Study design and setting**

This present study was conducted using the grounded theory approach. The study population includes key experts in the field of traffic and road safety, such as the Ministry of Health and Medical Education, Traffic Police, Ministry of Roads and Urban Development, Ministry of Industry, Road Safety Commission, legal Medicine Organization, Society of Traffic Thinkers, Municipalities, and private organizations involved in road safety.

**Participants**

Participants were included road safety experts and researchers, like epidemiologists in the area of road safety, disaster and emergency health, and traffic road safety specialists and accountable organizations including the police, Ministry of Health and Medical Education, Emergency Organization, Ministry of Roads and urban planning, the road safety commission, and the community of traffic thinkers. Participant selection was used in a targeted manner. On the other hand, the selection of participants by snowball method and maximum variety sampling was used to select participants and with the progress of data collection, the number of participants was determined based on saturation criteria. Inclusion criteria were willingness to conduct interviews as well as having experience in research. To select key informants and researchers, they referred to organizations and research centers related to the subject and collected data from them. 

**Data generation**

In this study, in depth interview method was used to collect data. To perform this, first four unstructured interviews were conducted. At this stage, the main concepts for the interview guide were obtained. Then a number of 15 semi-structured interviews were conducted using the interview guide. The prepared guide was provided to understanding of the process of Vision Zero implementation, the influencing conditions of the process, action and interaction strategies and process results. To ask questions, first opening questions, then introductory questions, and finally key question and probing questions were used.

Sample questions were included: What is your opinion about the establishment of Vision Zero in Iran? Then, when the data were analyzed over time and the initial concepts were extracted and the interview axes were formed, in-depth semi-structured interviews were conducted. To conduct interviews with the participants, after coordinating with them by telephone, the researcher was present at the time and place determined by the participants. Then, after explaining the objectives of the study and the confidentiality of the information, the participants' consent was obtained for the interview. The interview was conducted by the principal researcher (HS) in Persian. The content of the interview with the participants was recorded by two digital audio recorders. Furthermore, during the interview, Memo method was used to ask questions in subsequent interviews. At the end of the interview, participants were asked to contact the researcher if they needed more information. Interviews ranged from 21 to 51 minutes with an average of 33 minutes. The audio files were typed immediately by Word Office 2010 after the listening to interviews several times. 

**Data analysis**

Data analysis was performed after listening to the audio file few times and reconciling the transcribed content with the recorded audio. To data analysis, the Strauss and Corbin method was used. This qualitative research method is very useful when the researcher's goal is to explore a new field or to identify a known field from a new perspective.^26^ Data analysis is based on three basic steps including open coding to analyze the initial data, axial coding to explore relationships between the concepts, and finally selective coding to inferring a central core category and the research theory. During open coding, the research team focused on constructing concepts. For this purpose, after repeated study of the interviews, the concepts of the extracted data were identified. 

After any interviews and during data analysis, the constants comparison method was used to compare similarities and differences in data. Then the principal concepts were merged and new categories were formed. Concepts and categories were classified as causal conditions, intervening conditions, contextual conditions, action-interaction strategies and finally consequences based on the Strauss-Corbin model.

**Trustworthiness**

To create reliability in this study, the strategies recommended by Guba et al.^27^ including credibility, confirmability, transferability, dependability and reflexivity were used. Prolong Engagement of the researcher’s subject was used to create credibility. Peer check was done through the research group by holding meetings and discussing the data and analysis between the researchers themselves as well as with the experts. Moreover, recording the interviews / and converting them into written transcripts, reviewing interview summaries, and analyzing the obtained data and codes by member check and considering the feedbacks on their comments and ideas were done. When the extracted codes were not approved by the participants, the necessary explanations were acquired from them and the coding method was reviewed. Confirmability indicates the relationship between data and the used resources. Adherence to this criterion emphasizes that the results of the study have nothing to do with the knowledge of the researcher.^28^ The confirmability was done by the principal researcher (HS) using reviewing and collecting the ideas and thoughts of other researchers and applying the documentation of related studies. In this study, confirmability was reviewed by observers. To do this, the attained interviews, codes and categories were reviewed by several experts in the field of study. To achieve dependability, audit trail, stepwise replication, code-recode strategy and peer examination were used.^29^ To ensure transferability, comprehensive data description, and theoretical / purposive sampling were used.^27,29^ Moreover, the field of interviews, codes and concepts extracted by the research team and other professional colleagues of the field of qualitative research, were examined. Using Maximum variation sampling, researchers were able to gather a wide range of opinions, and different interpretations.^29^ Reflectivity of the process is the critical reflection on oneself as a researcher (biases, preferences, preconceptions) and the research relationship (relationship with the respondent and how this relationship affects participants' responses to questions).^30^ In this study, researchers have tried to describe the reflective notes for the interview, the setting and the aspects of the interview that were mentioned during the interview, while recording the audio tape and analyzing it. Also, the reflective notes of this study included the researcher's mental responses to setting and the relationship with the interviewees. Therefore, interviews, observations, focus group discussions, and all analytical data were completed using reflective notes.

## Results

**Figure 1:**
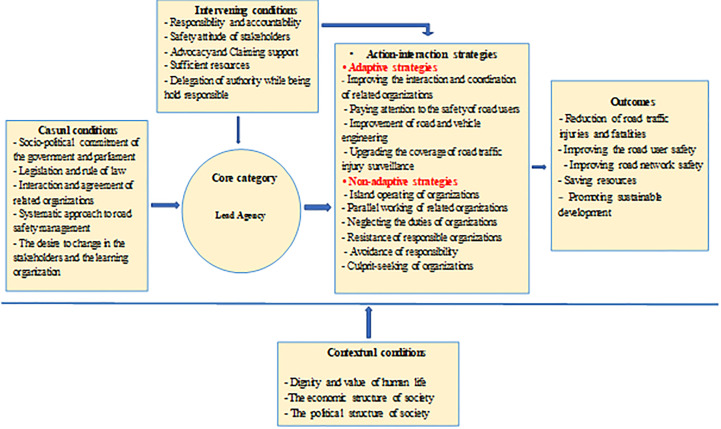
The model of Vision Zero implementation in Iran.

In this study, 19 interviews were conducted with 17 participants. The age range of the participants was 35-59 years and the work experience of the participants was at least 10 years (Table 1). A total of 1170 initial codes were extracted from the interviews. After summarizing and deleting similar codes, their number was reduced to 314 and then the codes were used to create categories and sub-categories. Finally, based on data analysis, a total of 4 main categories and 13 sub-categories were obtained. According to the participants, the lead agency recognized as the main variable. Other variables were classified as causal conditions, intervening conditions, action-interaction strategies, and consequences (Figure 1).

**Table 1:** Demographic characteristics of the participants in the study of the Vision Zero implementation model in Iran.

Participants		Frequency	Percentage
**Sex**	**Male**	13	76.47
	**Female**	4	23.52
**Age**	**30-40**	3	17.64
	**41-50**	9	52.95
	**Over 50**	5	29.41
**Work experiences (Year)**	**1-10**	3	17.64
	**11-20**	9	52.95
	**21-30**	5	29.41
**Levels of interview**	**Academic specialists**	6	35.29
	**Middle managers/stakeholders**	6	35.29
	**Top managers/stakeholders**	5	29.42

**Core category**

To ensure the association between the core category and others concepts, the role of core variable was investigated in all extracted concepts, subcategories, and categories. In this study, the lack of a lead agency was recognized as a variable that affects by all concepts in the Vision Zero implementation process. In order to implement Vision Zero in Iran, the lack of a lead agency was reported as the main concerns of the participants in this study. From the participants' perspective, the most important reason for the existence of a lead agency is that such an organization must exist in order to have a sight or vision. In the area of the road safety the main approach is reducing collisions before improving the safety or even prevention and this is because of the lack of a proper lead agency. According to most participants, before doing anything, the organization and the lead agency must be identified.

"We do not have a lead agency in the country, a lead agency, that manages this issue in the country, does not exist in our country, there is not any leadership unity that monitors all activities and policies, and there isn’t a specific person with a high authority to lead the agency "(P4)

**Causal conditions**

According to the participants, the most important factor influencing the establishment of the lead agency is the socio-political commitment of the government and parliament, legislation and lawfulness, as well as a systematic approach to road safety management. According to them, the establishment of a lead agency requires strong socio-political support from the government and parliament.

"We need to be supported by the government, the government and the parliament must be committed to this" (P4) ... "If the lead Agency is approved in the parliament, it will be very helpful in establishing a Vision Zero" (P12).

Furthermore, most participants saw the lack of adequate rules and legislation as a barrier to establishing a lead agency. According to them, without integrated rules and regulations and approved by all involved organizations, an effective and successful lead agency does not exist.

"The existence of a lead agency must become law, have legal functions and act lawfully. Without legislation, one cannot expect an efficient Lead Agency." (P15) To create such an organization with this level of caution, there is a need for upstream rules. "(P9)

Additionally, according to the participants, the traditional approach to road safety was one of the factors influencing the lead agency, which is very important in the implementation of Vision Zero. From their point of view, the country's approach to the prevention and management of road safety is not systemic and is individual and traditional. According to them, it is necessary to create a systematic organization to take a systemic approach to the prevention and management of RTCs.

"Our approach to road safety is not systemic. We do not have a systemic view either" (P5) .... The lead agency and Vision Zero implementation needs a systematic and comprehensive view of road safety"(P4)

Another causal condition in creating a lead agency was the unwillingness to change the approach among officials and stakeholders. According to the participants, the stakeholders are not willing to change their approach. According to them, all levels involved in road safety management must be ready for change in order to create a powerful and efficient management organization and, as a result, to implement Vision Zero well.

"Officials and stakeholders are reluctant to change their approach," (P4) ... All trustees and stakeholders should be involved in the change and prepare themselves for the change. (P4) ... Without creating change and willingness to change the approach, the organization cannot be an effective lead agency (P17).

**Intervening conditions**

From the most of the participants’ perspective, the responsibility and accountability of the stakeholders was one of the intervening factors in creating the lead agency. According to them, without accepting responsibility and proper accountability, one cannot have a real lead agency. Inadequate responsibility of organizations related to road safety management will lead to their mismanagement. Relevant organizations refuse to accept their responsibilities, and each blames the other. In cases where the occurrence of an accident is accompanied by mass casualty and public opinion is influenced, organizational projection and non-acceptance of responsibility become more apparent.

"Every organization defends itself and there is no final participation. Organizations are not responsible enough and do not have enough accountability." (P1) ... "Unless the relevant organizations are not responsible for their duties, they cannot be responsible and accountable to create a Lead Agency. "(P13)

Another intervening factor in the creation of the lead agency was the safety attitude of the stakeholders. From the participants' point of view, safety is not the focus of activities among officials and stakeholders, and they do not prioritize safety. In Iran, little attention is paid to the issue of safety, and the attitude of safety stakeholders has been overwhelmed by economic and political interests. Lack of sufficient attitude to safety causes a lack of proper understanding of preventive and control measures in the management of road safety and an obstacle to the need to establish a lead agency and the implementation of Vision Zero.

"Most officials do not have an attitude towards safety. There should be an integrated safety attitude by a Lead Agency to prevent traffic accidents with a priority of safety (P12) ... Most trustees have a political-economic view rather than a safety view ... (P8).

**Contextual conditions**

In this study, the dignity and value of human life, user-based transportation network, economic structure of society, and political structure of society, affect the core variable, and thus the action/interaction strategies.

In perspectives of the participants, the dignity and value of human life was one of the contextual factors in creating a lead agency and implementation of a Vision Zero in Iran. According to them, the dignity and value of human life are less important than the transportation system, and the transportation system places little value on human life. Without considering human ethics in managing road safety, it is not possible to have a proper understanding of human beings and their characteristics and behaviors.

"Our approach to human beings is not based on ethics. We do not look at people as they are valuable .... (P4)" In Iran, the ethical aspects of traffic accidents are not well observed. Stakeholders must adhere to ethics and the value and status of human dignity. "(P8)

Furthermore, according to participants’ viewpoints, the economic and political conditions of the society are among the contextual conditions influencing the implementation of a Vision Zero. According to them, the economic and political conditions of the society are effective in prioritizing the establishment of a lead agency and the implementation of Vision Zero.

"The implementation of Vision Zero should be done according to the economic and political context of the society. Economic conditions can affect the prioritization of the implementation of Vision Zero ... "(P17) ..." Politically, the government has not been able to provide the basis for the establishment of a lead agency. The government's priorities are different "(P14).

**Action / interaction strategies**

Based on the analysis of the extracted data, adaptive strategies include improving the interaction and coordination of related organizations, paying attention to road user safety, improving of road and vehicle engineering, and improving traffic surveillance system coverage, and non-adaptive strategies including island operations of Organizations, parallel work of related organizations, neglecting the duties of organizations, resistance of responsible organizations, avoidance of responsibility, arrogance and culprit-seeking of organizations were obtained.

Based on the experiences of the participants, if there is a lead agency, the interaction and coordination of the organizations will increase and more attention will be paid to road safety, especially for road users. It is also one of the strategies to interact with the lead agency through the promotion of road and vehicle engineering.

"… If we have a lead agency, we can better interact and coordinate between organizations. Also, road safety and road users become more important ...." (P15) … ". Vehicles can go towards the implementation of Vision Zero "(P17).

According to the participants, if there is a lead agency, the coverage of road traffic injury surveillance has been improved, which will affect the consequences. According to them, the road traffic injury surveillance system in interaction with other strategies can improve safety and success in the implementation of Vision Zero.

If the lead agency be existing, what is being done is that in addition to creating traffic accident surveillance, more coverage will be given and improved (P14). It has the basis of Vision Zero ". (P7)

Based on the experiences of the participants, in the absence of a lead agency, the organizations do not have enough interaction and coordination and act as an island. There is also parallel work in the tasks of organizations and each organization assigns responsibility to other organizations and blames them. In other words, the responsible organizations have resisted in terms of accountability and responsibility and blame other organizations.

"When the organization is not a leader, each organization builds its own and there is not enough coordination and they act as an island" (P7) ".... Organizations shifting blame on each other and do not fulfill their duties and take a stand (P11).

**Outcomes**

Based on the obtained data, effective road safety management, improving road user safety, improving road network safety, saving resources, reducing traffic accidents mortality, promoting sustainable development are the consequences of adaptive interaction strategies. From the participants' point of view, improving the interaction and coordination of related organizations improves road safety management by creating a leading organization. They also believed that improving road user safety, improving road and vehicle engineering, and improving traffic accident surveillance coverage would improve the transportation network, reduce deaths from RTCs, and ultimately promote sustainable development.

"If we have a lead agency, it will certainly improve road safety and reduce mortality as a result of better coordination" (P16). "The existence of a lead agency saves resources and with the implementation of Vision Zero, efforts should be made for sustainable development" (P14).

## Discussion

This study was conducted for the first time in Iran with the aim of explaining the Vision Zero implementation model using the qualitative grounded theory. The results of the current study show the association between different conditions affecting the Vision Zero implementation as a model. The results of the study showed that the lack of a lead agency is the main concern in the process of implementing Vision Zero. Lack of lead agency leads to lack of coordination and cooperation of stakeholders and lack of responsibility to promote safety among stakeholders.^31^ In LMICs, the structure of the transportation system may experience a lack of institutional performance among key safety actors. Therefore, it is necessary to establish an intra-organizational and inter-organizational evaluation framework that includes all processes, incidents, dependencies and causality among road safety stakeholders.^24^ Establishing a lead agency for better road engineering and more accurate enforcement of traffic laws with economic and non-economic penalties for unsafe traffic behaviors can improve road safety.^32^ In Iran, the road safety commission council (RSC) was established in 2003 as the main stakeholder of road safety; However, due to the lack of effective institutional management by the lead agency in several managerial functions, Iran has little chance to implement effective interventions in road safety and achieve the desired results.^24^

In this study, the socio-political commitment of the government and parliament, legislation and the rule of law, the systematic approach to deaths due to RTCs, and the tendency to change trustees were among the causal factors influencing the establishment of a lead agency in the Vision Zero model. Political commitment plays an important role in the management of RTCs^33^ and is considered as one of the Vision Zero operational strategies.^16^

Political commitment by the government at the highest level is essential to improve road safety. To achieve this, road safety leaders not only need to extend evidence-based road safety programs, but also need to support solutions that reflect an understanding of political limitations such as the electoral cycle.^11^ Political support is needed not only with a commitment to rules and regulations, but also with a commitment to the budget, with a long-term perspective.^11^ Community leaders who are generally committed to Vision Zero can contribute to the successful development, implementation and evaluation of Vision Zero.^34^

To ensure this, the managers not only need to develop evidence-based road safety programs, but also need to support strategies that reflect an understanding of political constraints such as the election cycle.

The systemic approach pays special attention to weakness and intrinsic characteristics and tolerance of human. This approach considers road safety designers as responsible for improving safety and preventing injuries caused by RTCs and states that responsibility should be distributed among system administrators and road users.^4^ Vision Zero's view is basically system-oriented and has a holistic and systemic view.^4^ In the traditional approach, instead of focusing on the cause of the damage, they focus on human error, poor vehicle design, and the road environment. In many LMICs, most preventive activities target road user behavior, which is usually countered by training and implementation. However, while safe user behavior is one of the important components, changing such behaviors should not be focused solely on education and rules.^35^

Another important factor influencing the establishment of the lead agency and the implementation of Vision Zero is legislation and rule of law. Legislation is one of the important principles of road safety management and Vision Zero is no exception to this basic principle.^8^ Establishing a lead agency requires comprehensive and strong laws from the government and parliament as stakeholders of the system. From a Vision Zero point of view, both the road user and the system administrators are responsible for enforcing the rules; But the ultimate responsibility for its implementation lies with the stakeholders.^36^ Weak rules lead to its incorrect implementation by the road user and ultimately the system as a whole.^37^

Another factor influencing the implementation of the Vision Zero in this study is the tendency to change the stakeholders and officials. This study revealed that stakeholders are reluctant to change their approach to safety management. In their view, their approach to managing road safety is correct and effective enough. From the perspective of Vision Zero, changes must be made in order to control and manage road safety.^8^ Suppliers and legislators must do their best to ensure the safety of users and must be prepared to change to achieve road safety goals.^11^

In this study, factors such as responsibility and accountability, safety attitude of stakeholders, support and demand, sufficient resources, sufficient authority of related organizations have been as intervening conditions in creating a lead agency and thus implementing a Vision Zero. Vision Zero emphasizes on change in road safety responsibility and expresses that the system designers are ultimately responsible for whole aspects of the road safety system.^10,16,38,39^ On the other hand, the demand for road safety in all dimensions, including child safety, pedestrian safety, safety of vulnerable users should be strengthened in all road users, especially vulnerable users. This is the focus of the UN slogan "Speak Up to Save Lives" in its latest slogan for road safety.^40^ If road users are unable to follow the rules, system designers must take further steps to prevent death or serious injuries.^10,11,38^

In this study, safety attitude was another intervening factor in creation of lead agency. Philosophy and attitude of safety is one of the basic principles of Vision Zero.^4^ According to Vision Zero, the transportation system must be designed to be responsive to human errors while preventing death or serious injuries,^8^ which means a philosophy and safety approach in Vision Zero. In order to establish and implement a Vision Zero, a culture and safety attitude must be properly embedded in the transportation system.^16,41^

Advocacy was another intervention factor in this study. Advocacy removes major obstacles to the implementation of proven policies and actions .^42^ Efforts to introduce proved road safety measures stand in opposition by groups with minority interests. Thus, experts and stakeholders must act as a powerful lobby for change.^42^ Besides, in the field of advocacy, NGOs participate in a variety of activities, from general awareness raising to lobbying for a specific legislative change.^43^

In this study, improving the interaction and coordination of related organizations, improving the safety of road users, promoting road and vehicle engineering, and improving the coverage of road traffic injury surveillance are among the adaptive strategies that are formed with the existence of the lead agency. Studies have shown that the existence of a lead organization brings about coordination and cooperation of stakeholders and their responsibility in advancing stakeholder safety.^31^ In the absence of a leading authority, inconsistent strategies emerge, including the island operating of organizations, the parallel work of related organizations, the neglect of the tasks of organizations, the resistance of responsible organizations, the avoidance of responsibility, arrogance and culprit-seeking of organizations. Currently, in Iran, tasks and roles related to road safety management are carried out by several organizations. Therefore, there is no clear structure and inter-organizational coordination in road safety management in Iran.^44^ Also, the results of a study showed that the lack of a traffic accident surveillance system is one of the most important road safety challenges in Iran.^31^ Traffic accident surveillance system is effective in formulating strategies, planning, implementing interventions, and allocating resources in preventing injuries.^45^ Hence, in order to implement Vision Zero in Iran, a comprehensive system of traffic injury surveillance is needed. 

Finally, the consequences of creating a leading agency and implementing Vision Zero include reducing road traffic fatality, effective management of traffic accidents, improving road user safety, improving road network safety, saving the resources, and promoting sustainable development.

## Conclusion

The implementation of the Vision Zero is complex and exposed to many determinants that are themselves influenced by other factors. The final model of this study provides a useful and practical model for implementing a Vision Zero by combining the extracted concepts and components as well as presenting proposed strategies and solutions and finally drawing the desired outcomes. Creating a lead agency with external coordination through political support and legislation and changing the approach of traffic accidents can be effective in implementing the Vision Zero. Also, improving the safety attitude of the stakeholders and changing their approach through training and seeking support from various organizations related to road safety is effective in creating a lead agency and implementing a Vision Zero. In addition, in order to implementation of the model, it is very important to pay attention to the economic, political and ethical contextual factors towards human beings.

**Acknowledgements**

We wish to thank all the participants who helped us in this study.
